# The Role of Macrophage Migration Inhibitory Factor (MIF) in Ultraviolet Radiation-Induced Carcinogenesis

**DOI:** 10.3390/cancers2031555

**Published:** 2010-08-09

**Authors:** Tadamichi Shimizu

**Affiliations:** Department of Dermatology, Graduate School of Medicine and Pharmaceutical Sciences, University of Toyama, Sugitani, 930-0194, Toyama, Japan; E-Mail: shimizut@med.u-toyama.ac.jp; Tel.: +81-76-434-7305; Fax: +81-76-434-5028

**Keywords:** skin, macrophage migration inhibitory factor, apoptosis, p53, cancer

## Abstract

Ultraviolet (UV) radiation is the most common cause of physical injury to the skin due to environmental damage, and UV exposure substantially increases the risk of actinic damage to the skin. The inflammatory changes induced by acute UV exposure include erythema (sunburn) of the skin, while chronic exposure to solar UV radiation causes photo-aging, immunosuppression, and ultimately, carcinogenesis of the skin. After skin damage by UV radiation, the cells are known to secrete many cytokines, including interleukin (IL)-1, IL-6, tumor necrosis factor (TNF)-α. and macrophage migration inhibitory factor (MIF). MIF was originally identified as a lymphokine that concentrates macrophages at inflammatory loci, and is known to be a potent activator of macrophages *in vivo*. MIF is considered to play an important role in cell-mediated immunity. Since the molecular cloning of MIF cDNA, MIF has been re-evaluated as a proinflammatory cytokine and pituitary-derived hormone that potentiates endotoxemia. MIF is ubiquitously expressed in various tissues, including the skin. Recent studies have suggested a potentially broader role for MIF in growth regulation because of its ability to antagonize p53-mediated gene activation and apoptosis. This article reviews the latest findings on the roles of MIF with regard to UV-induced skin cancer.

## 1. Introduction

The effects of sunlight have fascinated researchers for decades because nearly every living organism on earth is likely to be exposed to sunlight, including its ultraviolet (UV) fraction. UV radiation is divided into 3 subtypes, UVA (320–400nm), UVB (280–320nm) and UVC (200–280nm), each of which has distinct biological effects. Although UVC is blocked by stratospheric ozone, UVB (1–10%) and UVA (90–99%) reach the surface of the earth and cause skin damage [1]. The increased risk of skin damage from UV light has recently been linked to the decrease in stratospheric ozone. The health risks associated with ozone depletion are caused by enhanced UVA irradiation in the environment and increased penetration of the UVB light [2,3]. The skin is the body’s main interface with the environment, and is frequently exposed to UV light. Exposure to UV radiation substantially increases the risk of actinic damage to the skin. 

UV irradiation leads to various acute deleterious cutaneous effects, including sunburn and immunosuppression, as well as long-term consequences such as premature aging and the potential development of skin cancers [4]. In recent years, there has been increased interest in the contribution of UVA to skin carcinogenesis [5]. However UVB has been demonstrated to be a causal factor for basal cell carcinoma, squamous cell carcinoma, and lentigo maligna in epidemiological and experimental studies, and UVB exposure has been shown to induce the superficial spread of melanoma in humans and other animals [6]. Chronic UVB-induced inflammatory responses, immunosuppression, and direct DNA damage can be correlated with skin tumor formation [7,8]. Furthermore, the inability to adequately repair DNA after UVB irradiation can result in the formation of skin cancers [9].

## 2. UV-Induced Inflammatory Cytokines in the Skin

Epidermal cells are considered to be the major target of UVB radiation, as the vast majority of UVB is absorbed within the epidermis. There is emerging evidence that keratinocytes participate in cutaneous inflammatory reactions and immune responses by producing a variety of cytokines. UV irradiation may trigger cutaneous inflammatory responses by stimulating epidermal keratinocytes to produce biologically potent cytokines such as interleukin (IL)-1 [10,11], IL-6 [12], and tumor necrosis factor (TNF)-α [13]. Once expressed, TNF-α affects a variety of cell types. It increases MHC class I expression on endothelial cells and dermal fibroblasts; induces the production of IL-1α [14]; increases the expression of adhesion molecules, including ICAM-1, VCAM-1 and E-selectin; and it promotes the formation of sunburn cells [15]. Furthermore, these cytokines are involved not only in the mediation of local inflammatory reactions but also play discrete roles in tumor promotion [16].

## 3. Macrophage Migration Inhibitory Factor (MIF)

Macrophage migration inhibitory factor (MIF) was originally identified as a lymphokine that concentrates macrophages at inflammatory loci. MIF is a potent activator of macrophages *in vivo* and is considered to play an important role in cell-mediated immunity [17,18]. Since the molecular cloning of MIF cDNA [19], MIF was reevaluated as a proinflammatory cytokine and pituitary-derived hormone that potentiates endotoxemia [20]. Subsequent work has shown that T cells and macrophages secrete MIF in response to glucocorticoids, as well as upon activation by various pro-inflammatory stimuli [21]. MIF has been reported to be primarily expressed in T cells and macrophages; however, recent studies have revealed this protein to be ubiquitously expressed in various cells, thus indicating that it has a more far-reaching non-immunological role(s) in a variety of pathologic states [22,23,24,25,26]. Furthermore, MIF has broad action on induction of matrix metalloproteinases [27], glucocorticoid-induced immunomodulator [28], D-dopachrome tautomerase activity [29], innate immunity relevance to Toll-like receptor 4 [30] and is a crucial effector of hypoxia-inducible factor 1α that delays senescence [31]. It is known that MIF binds to the CD74 extracellular domain, a process that results in the initiation of a signaling pathway in a CD44 dependent manner [32,33]. Recently, it is demonstrated that CD74 forms functional complexes with CXCR4 that mediate MIF-specific signaling [34]. 

In the skin, MIF is expressed in the epidermal keratinocytes and fibroblasts [35,36]. Keratinocytes are capable of enhancing MIF production in the skin after UV radiation [36,37]. MIF is known to play an important role in the skin with regard to inflammation, the immune response, cutaneous wound healing [38] and skin disease, such as atopic dermatitis [39,40]. In addition, skin melanoma cells express MIF mRNA and produce MIF protein [41]. The expression of MIF mRNA and the production of MIF protein have been shown to be substantially higher in human melanoma cells than in cultured normal melanocytes. Therefore, MIF functions as a novel growth factor that stimulates the uncontrolled growth and invasion of tumor cells [41,42]. It is reported that MIF-deficient macrophages suppresses enhanced apoptosis and restores proinflammatory function. MIF inhibits p53 activity in macrophages via an autocrine regulatory pathway, resulting in a decrease in cellular p53 accumulation and subsequent function [43]. Recent studies have therefore suggested a potentially broader role for MIF in growth regulation because of its ability to antagonize p53-mediated gene activation and apoptosis [44,45].

## 4. MIF Inhibits p53-Dependent Apoptotic Processes Following UV Radiation

Apoptosis and enhanced DNA repair are both mediated by the p53 tumor suppressor [46]. The most commonly known UV-induced mutations occur in the p53 gene (TP53), and chronic UV exposure can increase the occurrence of TP53 mutations, leading to dysregulation of apoptosis, and the initiation of skin cancer [47]. UV-induced DNA lesions can lead to cell cycle arrest, DNA repair, and apoptosis if the DNA damage is beyond repair. UV radiation induces p53, which in turn leads to increased p21 synthesis, which arrests the cell cycle in the S1 phase, enabling DNA repair to occur. However, p53 can also participate in the initiation and regulation of the DNA repair process. In response to UV exposure, the protein bax is activated, which induces apoptosis and leads to the safe elimination of damaged cells. Therefore, it is important that apoptosis is induced immediately after UV irradiation. Any dysregulation in p53 signaling, especially with regard to its transcription of cell cycle regulatory and DNA damage response proteins, can result in cellular transformation. Recent evidence suggests that the pathways responsible for the removal of UV-mediated DNA damage can be modulated by inflammatory cytokines, including MIF.

**Figure 1:**
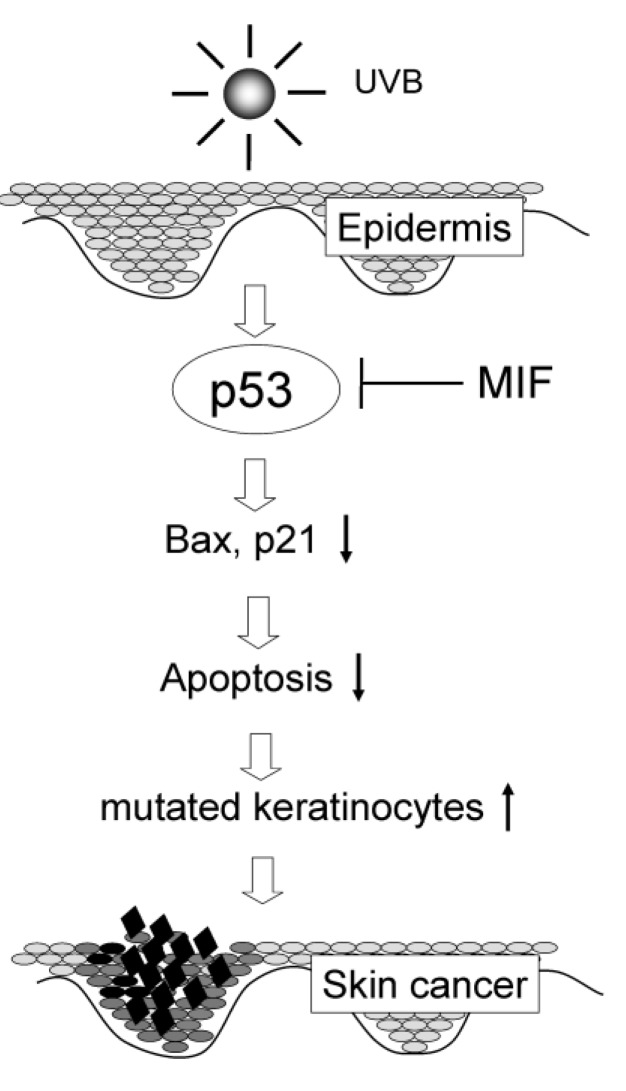
MIF inhibits p53-dependent apoptotic processesafter UVB exposure. UVB exposure enhances MIF production, whichinhibit the p53-dependent apoptotic processes by blocking the relevant expression of apoptosis-regulatory genes p53, p21, and bax, thereby inducing photocarcinogenesis in the skin.

MIF is a cytokine that not only plays a critical role in several inflammatory conditions but also inhibits the p53-dependent apoptotic processes [48]. Studies indicate that MIF treatment can overcome p53 activity and inhibit its transcriptional activity [37,49]. Recently, it has been demonstrated that after chronic UVB irradiation, an early onset of carcinogenesis and a higher tumor incidence were observed in transgenic (Tg) mice overexpressing MIF mice compared to wild-type (WT) mice [50]. In addition, the UVB-induced apoptosis of epidermal keratinocytes was inhibited in the MIF Tg mice. Significantly fewer terminal deoxyribonucleotidyl transferase (TDT)-mediated dUTP-digoxigenin nick end labeling (TUNEL)-positive cells were detected in MIF Tg mice than in WT mice. There was also a decrease in the expression of apoptosis-regulatory genes (*i.e.*, p53 and bax), in the MIF Tg mice after UVB irradiation (Figure 1). A previous study has confirmed that similar protective effects were observed in the corneas of MIF Tg mice in response to acute UVB light [51]. MIF is upregulated by UVB irradiation in the mouse cornea, and MIF Tg mice showed fewer apoptotic corneal cells. Similarly, other studies reported that MIF-deficient mice showed a significant increase in p53 activity and reduced tumor incidence compared to WT mice after acute and chronic exposure to UVB radiation, respectively [52]. MIF has also been shown to have an inhibitory effect on UVB-induced photo-damage by blocking the expression of apoptosis-regulatory genes [50].

## 5. MIF Exerts Pro-neoplastic Activity after UV Radiation

In addition to decreasing protective DNA damage repair, MIF also exhibits direct pro-neoplastic activity. In many tumor cells and pre-tumor states, for example, in prostate [53], colon [54], and hepatocellular cancers [55]; adenocarcinomas of the lung [56]; glioblastomas [57]; and melanomas [41], increased MIF mRNA levels can be detected. The pro-neoplastic role of MIF has been studied by several groups. Fingerle *et al*. reported that embryonic fibroblasts from MIF-deficient mice exhibit p53-dependent growth alterations, increased p53 transcriptional activity, and resistance to ras-mediated transformation [37,48,49]. Concurrent deletion of the p53 gene *in vivo* reversed the observed phenotype of cells deficient in MIF. *In vivo* studies showed that fibrosarcomas are smaller in size and have a lower mitotic index in MIF-deficient mice relative to their WT counterparts. The authors concluded that there is direct evidence for a functional link between MIF and the p53 tumor suppressor [37,48,49]. 

It has also been reported that using an anti-MIF antibody is effective for reducing tumor growth and neovascularization in lymphoma cells and vascular endothelial cells *in vivo* [26]. Consistent with this finding, anti-MIF antibodies were found to be effective for reducing tumor angiogenesis in melanoma cells [41]. This was demonstrated *in vitro* by using recombinant MIF in fibroblasts, where growth-factor-induced stimulation of these cells resulted in increased MIF concentrations, activation of the extracellular regulated kinase/mitogen-activated protein (ERK-MAP) kinase pathway, and a subsequent increase in cell proliferation [58]. In addition, it has been shown that exposure to TGF-β results in increased MIF expression in a colon cancer cell line [59]. Furthermore, other investigators observed an increase in the level of cytotoxic T lymphocytes following MIF inhibition achieved by using specific antibodies (REF), and the number of apoptotic tumor cells increased following MIF inhibition [60]. Tumors arising in the MIF-knockdown cells grew less rapidly and also showed an increased degree of apoptosis [61]. These findings, therefore, suggest that once keratinocytes acquire mutations by UVB-induced DNA damage, they may become malignant, and MIF may thus play a dual role in promoting the growth of these tumor cells and inhibiting their apoptosis.

## 6. MIF-Induced Skin Inflammation and Carcinogenesis

UVB stimulates the production of several proinflammatory cytokines, such as TNF-α, IL-1, and IL-6 in the skin, and these proinflammatory cytokines are considered to be closely related to the progression of UV-induced carcinogenesis [62]. For example, TNF-α is an essential cytokine responsible for tumor promotion in mouse skin. Tumor promotion by 12-*O*-tetradecanoylphorbol-13-acetate (TPA) on the skin of TNF-α-deficient mice decreased compared to that in WT mice [14]. Similarly, tumor promotion in IL-6-deficient mice was significantly decreased by TPA compared to that in the WT mice [14]. UVB-induced inflammatory responses, such as the production of cytokines and the infiltration of inflammatory cells, are clearly associated with the development of skin tumors. The inhibition of this inflammatory response via topical application of an anti-inflammatory drug inhibits the acute inflammatory responses after UVB exposure, and decreases tumor formation after chronic exposure [63]. 

MIF plays a direct role in both inflammation and tumorigenesis [64]. UVB exposure in MIF Tg mice resulted in greater leukocyte infiltration than in WT mouse skin [50]. Moreover, UVB irradiation enhances the expression of MIF in the epidermis, and MIF Tg mice showed higher levels of MIF mRNA expression after UVB exposure [37]. Once released, MIF acts as a proinflammatory cytokine to induce the expression of other inflammatory cytokines, including IL-1, IL-6 and TNF-α [65,66,67]. Therefore, intense inflammation in the skin in response to UVB irradiation was found to be correlated to the early onset of carcinogenesis and a higher incidence of tumors after chronic UVB exposure.

## 7. Conclusions

Chronic exposure to UV irradiation induces early-onset skin carcinogenesis, and many inflammatory cytokines are related to the induction and progression of UV-induced skin cancer. Chronic UVB exposure enhances MIF production, which may inhibit the p53-dependent apoptotic processes, thereby inducing photocarcinogenesis in the skin. This newly identified mechanism may contribute to our overall understanding of photo-induced skin damage, which ultimately results in carcinogenesis. 
